# General practitioners cannot rely on reported weight and height of children

**DOI:** 10.1017/S1463423618000713

**Published:** 2018-10-08

**Authors:** Janneke van Leeuwen, Marienke van Middelkoop, Winifred D. Paulis, Patrick J.E. Bindels, Bart W. Koes

**Affiliations:** 1 Department of General Practice, Erasmus MC, University Medical Center, Rotterdam, The Netherlands; 2 Department of Physical Therapy Studies, Rotterdam University of Applied Sciences, Rotterdam, The Netherlands

**Keywords:** childhood obesity, primary care, screening

## Abstract

*Aim:* The aim of this study is to investigate the differences between reported and measured weight and height for underweight, normal-weight, and overweight children, particularly in a general practitioner setting. *Background:* Screening, signaling, and treatment of childhood obesity by the general practitioner depends on accurate weight and height measurements.*Methods:* Data on reported and measured weight and height from a cohort including 715 normal-weight and overweight children aged 2–17 were used. Means of reported and measured weight and height were compared using the paired *T*-test. *Findings:* Of the 715 included children, 17.5% were defined as being underweight, 63.2% normal-weight, and 19.3% overweight according to direct measured height and weight. In the age group 2–8 years, parents of underweight children reported a significantly higher weight than measured weight [mean differences (MD) 0.32 kg (0.02, 0.62)], whereas parents of overweight young children reported a significantly lower weight [MD −1.08 kg (−1.77, −0.39)]. In the age group 9–17 years, normal-weight [MD −0.51 kg (−0.79, −0.23)] and overweight children [MD −1.28 kg (−2.08, −0.47)] reported a significantly lower weight than measured weight. *Conclusions:* General practitioners cannot rely on reported weight and height measures from parents and children. In case of suspected under- or overweight in children, it should be advised to measure weight and height in general practice.

## Introduction

Childhood obesity is a public health problem and its prevalence is increasing worldwide (James *et al.*, 2001).

Reported, rather than measured weight and height are often used to calculate body mass index (BMI) and to classify the child as being underweight, normal-weight, or overweight (Beck *et al.*, 2012). This method of data collection is quicker, easier and cheaper and therefor often performed in both clinical practice and research. However, parents presenting to health care providers may give inaccurate information on the child’s weight and height, since it has been shown that parents are likely to misperceive the weight status of their overweight child (Rietmeijer-Mentink *et al.*, 2013). As a result, children could be misclassified as being normal-weight rather than overweight or obese, which could lead to children missing out on proper and necessary treatment. Though, direct measurements of height and weight by a clinician are more-time consuming and more expensive.

General practitioners (GP) in the Netherlands are often the first health care provider of children and therefore play an important role in screening and signaling childhood obesity (Paulis *et al.*, 2017). The question arises whether the GP can rely on reported measurements by parents and children themselves or should children be measured during consultation at the GP? Therefore, this study aims to investigate the accuracy of reported weight and height in children aged 2–17 compared to direct measurements by the GP.

## Methods

### Study design

This study is a cross-sectional study using data from the DOERAK [“Determinants of (sustained) Overweight and complaints; Epidemiological Research among Adolescents and Kids in general practice”] cohort study. The study protocol has been published previously (Paulis *et al.*, 2012). The study has been approved by the Institutional Review Board of the Erasmus University Medical Center, Erasmus MC.

### Participants

Children aged 2–18 visiting their GP (or GP-trainee) between December 2010 and April 2013 were asked, during consultation, to participate in the study. This age range was used, since BMI-z scores can be calculated for children starting at age two and parents are legally responsible for their child up to the age of 18. Children were eligible to participate in the study if they/their parents had a basic understanding of the Dutch language, that is, to be able to give informed consent and fill out Dutch questionnaires. Children with mental or physical disabilities, with comorbidities affecting weight, and children visiting their GP with emergency problems were not eligible. If child and parent showed interest after receiving verbal information during consultation, the child’s weight and height were measured and recorded in the medical file, and contact information was sent to the research team. Study information and informed consent forms (and informed assent forms for children aged 12 and older) were then sent to the participants, where after the researcher contacted the family to answer possible questions and to investigate the willingness to participate. Both parents had to sign the informed consent (for children of all ages), and children aged 12 and older also had to sign the informed assent form. Children were formally included when informed consent forms (and if needed informed assent forms) were received.

### Data collection and measurements

After formal inclusion, the GP or GP trainee were approached to collect data on the child’s weight and height which was measured during consultation using calibrated scales and stadiometers. Measurements were performed by the GP or GP trainee who both followed the same study protocol (Paulis *et al.*, 2012).

The GP questionnaire was used to extract the participant’s gender and age. Baseline BMI-z scores were calculated from the measured weight and height, and weight status was determined using the international age and gender specific cut-off points (Cole *et al.*, 2000; Cole *et al.*, 2007). Children were then categorized in three different weight status groups: underweight, normal-weight, overweight/obese (from here on referred to as the overweight group).

Reported weight and height measures were collected from the baseline questionnaires which were filled out by parents of children aged 2–8, or children themselves (age 9 and older). From these reported weight and height measures, BMI-z scores, and corresponding weight status, were also calculated. The parent’s questionnaires were used to extract information on socio-economic status (SES) [based on net household income (<2000 euros/month, ⩾2000 euros/month)], ethnicity (both parents born in the Netherlands, at least one parent born in another country) and marital status reported by parents (parents living together, parents separated). Highest level of education in the household was categorized into three levels (up to lower secondary level, upper secondary level, at least bachelor level), based on the international standard classification of education (Centraal Bureau Voor De Statistiek, 2011).

### Statistical analysis

Baseline demographics were described for underweight, normal-weight, and overweight children using means (standard deviation) for continuous variables and frequencies (%) for dichotomous or categorical variables. Potential differences in baseline demographics between underweight and normal-weight, and overweight and normal-weight children were analyzed using the independent-samples *T*-test. In addition, potential differences in measured and reported height, weight, and BMI-z in the subgroups young (2–8 year) and older children (9–17 year), and boys and girls were analyzed using the paired *T*-test. The magnitude of the differences was determined using mean differences (MD) with 95% confidence intervals. Complete case analysis was used. *P*-values <0.05 were considered statistically significant. IBM SPSS statistics 12.0 was used for statistical analyses.

## Results

**Fig. 1:**
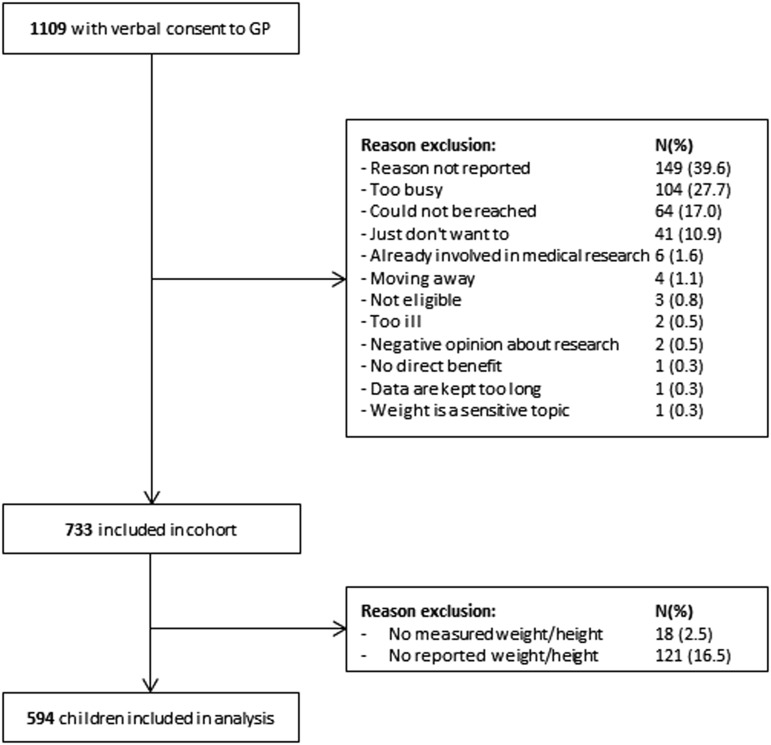
Flowchart of inclusion

Of the 1109 children that showed interest in study participation, 733 were included. Measured and/or reported weight and/or height was not available of 139 children who were excluded, and therefore 594 children were included in the present study (Figure 1). There were no significant differences in baseline characteristics between the excluded and included children.

At baseline, 18.2% of the children were defined as being underweight, 62.3% normal-weight, and 19.5% overweight according to direct measured height and weight (Table 1). The children in the underweight group were significantly younger than the normal-weight children (6.8 versus 8.3 years), while the overweight children were significantly older than the normal-weight children (9.3 versus 8.3 years).

**Table 1:** Baseline characteristics

Patient characteristics	Study population (*N*=594)	Underweight^a^ (*N*=108)	Normal-weight^a^ (*N*=370)	Overweight/obese^a^ (*N*=116)
Gender female [*N* (%)]	*N*=594 316 (53.2)	*N*=108 57 (52.8)	*N*=370 196 (53.0)	*N*=116 63 (54.3)
Age (years) [mean (SD)]	*N*=594 8.2 (4.0)	*N*=108 6.8 (3.9)^c^	*N*=370 8.3 (4.1)	*N*=138 9.3 (3.7)^c^
SES [*N* (%)]	*N*=541	*N*=98	*N*=340	*N*=103
Low (<2000 euros)	121 (22.4)	20 (20.4)	75 (22.1)	26 (25.2)
Middle/high (⩾2000 euros^b^)	420 (77.6)	78 (79.6)	265 (77.9)	77 (74.8)
Highest education in household [*N* (%)]	*N*=585	*N*=107	*N*=363	*N*=115
Low (up to lower secondary level)	99 (19.9)	19 (17.7)	61 (16.8)	19 (16.5)
Middle (upper secondary level)	238 (40.7)	37 (34.6)	147 (40.5)	54 (47.0)
High (at least bachelor level)	248 (42.4)	51 (47.7)	155 (42.7)	42 (36.5)
Ethnicity [*N* (%)]	*N*=569	*N*=107	*N*=351	*N*=111
Both parents born in the Netherlands	483 (84.9)	91 (85.0)	303 (86.3)	89 (80.2)
At least one parent born in another country	86 (14.5)	16 (15.0)	48 (13.7)	22 (19.8)
Marital status [*N* (%)]	*N*=582	*N*=107	*N*=362	*N*=113
Parents separated	93 (16.0)	16 (15.0)	56 (15.5)	21 (18.6)
Parents together	489 (84.0)	91 (85.0)	306 (84.5)	92 (81.4)

SES=socio-economic status.

a Weight status based on weight and height measures from general practitioner.

b More than 2000 euros monthly net income per household.

c Significantly different from normal-weight.

Analyses among the three weight groups showed that underweight children reported a significantly higher weight than measured [MD 0.58 kg (0.11, 1.05)] while overweight children reported a significantly lower weight than measured [MD −1.20 kg (−1.75, −0.65)]. In the normal-weight group, no significant differences were found. For height, no significant differences between reported and measured height were found for all weight groups.

The subgroup analyses among age groups showed that parents of underweight children aged 2–8 years, reported a significantly higher weight [MD 0.32 kg (0.02, 0.62)] and lower height [MD −1.01 cm (−1.69, −0.34)] than measured weight and height (Table 2). Parents of overweight children aged 2–8 reported a significantly lower weight [MD −1.08 kg (−1.77, −0.39)] and larger height [MD 1.09 (0.14, 2.04)] than measured weight and height. There were no significant differences between reported and measured weight and height for normal-weight children aged 2–8.

**Table 2:** Mean differences (MD) between reported and measured weight, height and BMI-z according to weight status and age group

	Weight (kg)				Height (cm)				BMI-z			
Weight status^a^ Age group	Reported mean (SD)	Measured mean (SD)	MD (95% CI)	*P*-value	Reported mean (SD)	Measured mean (SD)	MD (95% CI)	*P*-value	Reported mean (SD)	Measured mean (SD)	MD (95% CI)	*P*-value
Underweight^a^												
2–8 years	17.68 (3.99)	17.36 (4.06)	0.32 (0.02 –0.62)	0.03^b^	112.13 (13.96)	113.19 (13.31)	−1.06 (−1.87 –−0.25)	0.01^b^	−1.06 (1.56)	−1.67 (0.88)	0.60 (0.25 –0.97)	0.001^b^
9–17 years	34.82 (8.42)	33.65 (9.58)	1.17 (−0.25 –2.59)	0.10	150.35 (14.21)	149.46 (15.38)	0.90 (−1.78 –3.58)	0.50	−1.72 (1.10)	−2.06 0.84)	0.34 (−0.04 –0.71)	0.08
Normal-weight^a^												
2–8 years	21.87 (6.23)	21.76 (5.16)	0.11 (−0.38–0.60)	0.66	117.05 (14.53)	117.12 (13.84)	−0.07 (−0.83 –0.70)	0.87	0.01 (1.02)	0.10 (0.63)	−0.09 (−0.21 –0.03)	0.12
9–17 years	45.64 (11.40)	46.15 (11.64)	−0.51 (−0.79 –−0.23)	<0.001^b^	157.26 (13.39)	157.14 (13.20)	0.12 (−0.22 –0.47)	0.48	−0.14 (0.71)	−0.05 (0.67)	−0.10 (−0.15 –−0.04)	0.001^b^
Overweight^a^												
2–8 years	29.13 (9.02)	30.21 (8.89)	−1.08 (−1.77 –−0.39)	0.003^b^	124.45 (15.44)	123.36 (15.48)	1.09 (0.14 –2.04)	0.03^b^	1.41 (1.27)	1.98 (0.62)	−0.57 (−0.90 –−0.24)	0.001^b^
9–17 years	59.51 (16.08)	60.78 (15.65)	−1.28 (−2.08 –−0.47)	0.002^b^	156.88 (12.80)	156.87 (12.47)	0.01 (−0.67 –0.68)	0.99	1.77 (0.73)	1.92 (0.57)	−0.15 (−0.29 –−0.01)	0.03^b^

CI=confidence interval.

a weight status based on weight and height measures from the general practitioner.

b *P*-value <0.05.

Normal-weight [MD −0.51 kg (−0.79 , −0.23)] and overweight children aged 9–17 reported a significantly lower weight than measured weight [MD −1.28 kg (−2.08, −0.47)].

When looking at boys and girls separately, both overweight boys [MD −1.03 kg (−1.74, −0.31)] and overweight girls [MD −1.34 kg (−2.17, −0.51)] reported a significantly lower weight than measured. Boys aged 9–17 of normal-weight [MD −0.43 kg (−0.87, −0.001)] and overweight [MD −1.06 kg (−1.94, −0.18)], and girls aged 9–17 of normal-weight [MD −0.57 kg (−0.95, −0.19)] and overweight [MD −1.46 kg (−2.80, −0.12)] reported a significantly lower weight than measured. Parents of overweight girls aged 2–8 years reported a significantly lower weight than measured [MD −1.17 kg, −1.94, −0.40)].

Of the 109 children who were classified as underweight by the GP, 33 would be misclassified into the normal-weight group when using reported measurements, and one child into the overweight group. Of the children who were classified as overweight by the GP (total 116), 20 would be misclassified as normal-weight and four as underweight using self-reported measurements (Table 3).

**Table 3:** Weight status (mis)classification

	Based on self-reported data				
		Underweight [*N* (%)]	Normal-weight [*N* (%)]	Overweight [*N*(%)]	Total
Based on measured data	Underweight [*N* (%)]	74 (68%)^a^	33 (31%)	1 (1%)	108 (18%)
	Normal-weight [*N* (%)]	34 (9%)	322 (87%)^a^	14 (4%)	370 (62%)
	Overweight [*N* (%)]	4 (4%)	20 (17%)	92 (79%)^a^	116 (20%)
	Total	112 (19%)	375 (63%)	107 (18%)	594 (100%)

a Agreement on weight status between weight status based on self-reported data and based on measured data.

## Discussion

### Summary

Parents of underweight and overweight children aged 2–8 years reported a significantly higher and lower weight respectively, compared to measured weight. Normal-weight and overweight children aged 9–17 reported a significantly lower weight than measured. When looking at boys and girls separately, both normal-weight and overweight boys and girls aged 9–17 reported a significantly lower weight than measured. Parents of overweight girls aged 2–8 years reported a significantly lower weight than measured.

### Strength and limitations

The current study is one of the first to investigate the differences in reported and measured weight status in three different weight groups, split by age, in primary care. We were therefore able to investigate both how parents’ reported weight and height of young children differed from measured values, and how reported weight and height by older children differed from measured values.

By inviting every child visiting the GP during the inclusion period, we tried to minimize selection bias. However, when comparing our study population to the overall Dutch population, parents of included children in our cohort were more often born in the Netherlands (84 versus 79%) and more often highly educated (42 versus 32%) (Centraal Bureau voor de Statistiek, 2017). Since overweight and obesity is more prevalent in ethnic minorities and families of lower SES, selection bias in the current study may have led to an underestimation of the percentage overweight and obese children, and to an overestimation of underweight children (Stamatakis *et al.*, 2005). This is reflected by prevalence differences in underweight children of the current study (18.2%) when compared to the prevalence (1.6%) reported by the World Health Organization (WHO) (The World Bank, 1980) and reported by the Dutch Central Bureau of Statistics (5.7%) (Centraal Bureau voor de Statistiek, 2016). Therefore, we may have to be careful to generalize the results of the current study to a wider perspective. However, the differences in percentage underweight children between the current study and the WHO may be associated with the different cut-off points that were used to classify children as underweight, normal-weight or overweight (Cole *et al.*, 2000; Cole *et al.*, 2007; de Onis *et al.*, 2007). The WHO uses the WHO growth references, which rely on age–sex-specific BMI centiles or standard deviation scores to define the weight status cut-offs, while the current study used an international standard growth chart which was developed by The International Obesity Task Force, to enable global comparison (Cole *et al.*, 2000; 2007; de Onis *et al.*, 2007). However, since we were primarily interested in differences between reported and measured values within the different weight groups, we believe this did not significantly impact our results.

The size of our study sample was smaller than intended, which may have introduced a power problem (Paulis *et al.*, 2012). However, since we were able to show significant differences in reported and measured weight and height, we believe a larger study sample would not significantly change our results.

Lastly, when the GP measured the child’s weight status during consultation, the results were recorded into the medical file of the child, and not *per se* concealed from the parent/child. We believe enough time passed from this consultation to when the baseline questionnaire was filled out by parents or the child, so that the parent/child did not remember what the GP had measured during consultation. Furthermore, this procedure was identical for every included child. We therefore believe that this procedure did not have a significant impact on our results.

### Comparison with existing literature

Our findings are in line with previous literature (Beck *et al.*, 2012), showing that reported weight in overweight and obese young children is not accurate compared to measured values. Previous literature already showed that parents often misperceive the weight(status) of their overweight child (Rietmeijer-Mentink *et al.*, 2013). However, the current study showed that parents are also inaccurate in reporting weight of their underweight child. Not only parents, but also children aged 9–17 fail to accurately report their own weight (Sherry *et al.*, 2007). As a result, 32% of the underweight children and 21% of the overweight children in our study would be misclassified in the different weight categories.

Although no significant differences in SES between underweight, normal-weight, and overweight children were found, a trend is seen where overweight children come from families with a lower SES than underweight and normal-weight children. This is in line with other literature showing that obesity is more prevalent in children from ethnic minorities with a lower SES and level of education (Gishti *et al.*, 2014). However, in the current study, reported weight within a weight class was not significantly different between levels of SES, thus SES does not seem to influence the ability to accurately report weight.

### Implications for research and/or practice

According to international guidelines for primary care, the GP plays an important role in screening children on their weight status (Richardson *et al.*, 2013). In the Netherlands, school physicians also play a role in screening children, since they measure height and weight at age 5–6 and 10–11 years. However, these data are not transferred to GPs (Gemeentelijke Gezondheidsdienst, 2018). In the United Kingdom, a similar program is active, namely the National Child Measurement Programme (National Health Service, 2018). However, besides these set measurement times, no measured data are available and GP’s will rely on self-reported data. However, if a GP would rely on the reported weight measures of parents and children, part of these under- or overweight children would potentially be missed and therefore not receive proper treatment or referral. Thus, the GP cannot rely on reported weight and height measures from parents and children. In case of suspected under- or overweight in children, it should be advised to measure weight and height in general practice. However, it is known that GP’s find it difficult to discuss weight issues during consultation (Dettori *et al.*, 2009). Furthermore, research showed that although most GP’s are able to identify the underweight and obese children at the end of the spectrum, many are not able to correctly identify the weight status of children who are just underweight, or just obese (Gage *et al.*, 2012). Therefore, it could be argued that, to overcome these two issues, all children visiting the GP should be measured (at least yearly) as part of routine measurements so that accurate treatment and follow-up can be discussed during consultation.

## References

[ref1] Beck J, Schaefer CA, Nace H, Steffen AD, Nigg C, Brink L, Hill JO Browning RC (2012) Accuracy of self-reported height and weight in children aged 6 to 11 years. Preventing Chronic Disease 9, E119. 22742593 10.5888/pcd9.120021 PMC3457756

[ref30] Centraal Bureau Voor De Statistiek (2011) International Standard Classification of Education: Inpassen van het Nederlandse onderwijs in ESCED 2011. Voorburg, Netherlands.

[ref2] Centraal Bureau Voor De Statistiek (2016) Lengte en gewicht van personen, ondergewicht en overgewicht; vanaf 1981. Retrieved 16 February 2018 from http://statline.cbs.nl/StatWeb/publication/?DM=SLNL&PA=81565NED.

[ref3] Centraal Bureau Voor De Statistiek (2017) Bevolking; onderwijsniveau; geslacht, leeftijd en migratieachtergrond. CBS. Retrieved 16 February 2018 from http://statline.cbs.nl/StatWeb/publication/?VW=T&DM=SLnl&PA=82275NED&LA=nl.

[ref4] Cole TJ, Bellizzi MC, Flegal KM Dietz WH (2000) Establishing a standard definition for child overweight and obesity worldwide: international survey. BMJ 320, 1240–1243. 10797032 10.1136/bmj.320.7244.1240 PMC27365

[ref5] Cole TJ, Flegal KM, Nicholls D Jackson AA (2007) Body mass index cut offs to define thinness in children and adolescents: international survey. BMJ 335, 194. 17591624 10.1136/bmj.39238.399444.55 PMC1934447

[ref6] De Onis M, Onyango AW, Borghi E, Siyam A, Nishida C Siekmann J (2007) Development of a WHO growth reference for school-aged children and adolescents. Bulletin of the World Health Organization 85, 660–667. 18026621 10.2471/BLT.07.043497 PMC2636412

[ref7] Dettori H, Elliott H, Horn J Leong G (2009) Barriers to the management of obesity in children - a cross sectional survey of GPs. Australian Family Physician 38, 460–464. 19530380

[ref8] Gage H, Erdal E, Saigal P, Qiao Y, Williams P Raats MM (2012) Recognition and management of overweight and obese children: a questionnaire survey of general practitioners and parents in England. Journal of Paediatrics and Child Health 48, 146–152. 21535283 10.1111/j.1440-1754.2011.02058.x

[ref9] Gemeentelijke Gezondheidsdienst (2018) Jeugd & Gezondheid. Retrieved 5 June 2018 from https://www.vggm.nl/ggd/jeugd_en_gezondheid/wat_doet_de_jgz_4-18_jaar_/basisonderwijs.

[ref10] Gishti O, Kruithof CJ, Felix JF, Raat H, Hofman A, Duijts L, Gaillard R Jaddoe VW (2014) Ethnic disparities in general and abdominal adiposity at school age: a multiethnic population-based cohort study in the Netherlands. Annals of Nutrition and Metabolism 64, 208–217. 25300262 10.1159/000365022

[ref11] James PT, Leach R, Kalamara E Shayeghi M (2001) The worldwide obesity epidemic. Obesity Research 9 (Suppl 4), 228S–233S. 11707546 10.1038/oby.2001.123

[ref12] National Health Service (2018) National Child Measurement Programme. Retrieved 5 June 2018 from https://digital.nhs.uk/services/national-child-measurement-programme/.

[ref13] Paulis WD, Palmer M, Chondros P, Kauer S, Van Middelkoop M Sanci LA (2017) Health profiles of overweight and obese youth attending general practice. Archives of Disease in Childhood 102, 434–439. 27836827 10.1136/archdischild-2016-311404

[ref14] Paulis WD, Van middelkoop M, Bueving H, Luijsterburg PA, Van der wouden JC Koes BW (2012) Determinants of (sustained) overweight and complaints in children and adolescents in primary care: the DOERAK cohort study design. BMC Family Practice 13, 70. 22824438 10.1186/1471-2296-13-70 PMC3437208

[ref15] Richardson L, Paulis WD, Van Middelkoop M Koes BW (2013) An overview of national clinical guidelines for the management of childhood obesity in primary care. Preventive Medicine 57, 448–455. 23988494 10.1016/j.ypmed.2013.08.010

[ref16] Rietmeijer-Mentink M, Paulis WD, Van middelkoop M, Bindels PJ Van der wouden JC (2013) Difference between parental perception and actual weight status of children: a systematic review. Maternal & Child Nutrition 9, 3–22. 23020552 10.1111/j.1740-8709.2012.00462.x PMC6860751

[ref17] Sherry B, Jefferds ME Grummer-Strawn LM (2007) Accuracy of adolescent self-report of height and weight in assessing overweight status: a literature review. Archives of Pediatrics and Adolescent Medicine 161, 1154–1161. 18056560 10.1001/archpedi.161.12.1154

[ref18] Stamatakis E, Primatesta P, Chinn S, Rona R Falascheti E (2005) Overweight and obesity trends from 1974 to 2003 in English children: what is the role of socioeconomic factors? Archives of Disease in Childhood 90, 999–1004. 15956046 10.1136/adc.2004.068932 PMC1720119

[ref19] The World Bank (1980) Prevalence of underweight, weight for age (% of children under 5). Retrieved 16 February 2018 from https://data.worldbank.org/indicator/SH.STA.MALN.ZS?locations=NL.

